# The complete chloroplast genome sequence of medicinal plant: *Lepidium apetalum* (Brassicaceae)

**DOI:** 10.1080/23802359.2020.1790319

**Published:** 2020-07-15

**Authors:** Yi Liu, Chong Xiao

**Affiliations:** Hospital of Chengdu University of Traditional Chinese Medicine, Chengdu, Sichuan Province, PR China

**Keywords:** Chloroplast genome, phylogenetic analysis, Brassicaceae, *Lepidium apetalum*

## Abstract

*Lepidium apetalum* is a traditional Chinese medicine. The complete chloroplast genome sequence is 154,680 bp in length, with one large single-copy region of 83,787 bp, one small single-copy region of 18,013 bp, and two inverted repeat (IR) regions of 26,440 bp. It contains 128 genes, including 83 protein-coding genes, eight ribosomal RNAs, and 37 transfer RNAs. Phylogenetic tree shows that this species is sister to *L. sativum* and *L. virginicum.*

*Lepidium*, a genus, belongs to the Brassicaceae family, contains approximately 16 species in China (Zhou et al. 2001), which indicates that especially the seeds were esteemed for their medicinal properties. The seeds of *Lepidium apetalum* are aperient, diuretic, tonic, demulcent, carminative, galatogogue, and emmenagogue (Bansal et al. 2012). However, there are very few studies on *L. apetalum*, which greatly limit the development and utilization of *L. apetalum*. So far, the chloroplast genome of *L. apetalum* has not been reported. In this study, we assembled the complete chloroplast genome of *L. apetalum*, hoping to lay a foundation for further research.

Fresh leaves of *L. apetalum* were collected from Nanhuashan mountain (Zhongwei, Ningxia, China; coordinates: 105°38′E, 36°21′N) and dried with silica gel. The voucher specimen was stored in Sichuan University Herbarium with the accession number of QTPLJQ13383021. Total genomic DNA was extracted with a modified CTAB method (Doyle and Doyle 1987). First, we obtained 10 million high quality pair-end reads for *L. apetalum*, and after removing the adapters, the remained reads were used to assemble the complete chloroplast genome by NOVOPlasty (Dierckxsens et al. 2017). The complete chloroplasts genome sequence of *L. sativum* was used as a reference. Plann v1.1 (Huang and Cronk 2015) and Geneious v11.0.3 (Kearse et al. 2012) were used to annotate the chloroplasts genome and correct the annotation.

The total plastome length of *L. apetalum* (MT588298) is 154,680 bp, exhibits a typical quadripartite structural organization, consisting of a large single-copy (LSC) region of 83,787 bp, two inverted repeat (IR) regions of 26,440 bp and a small single-copy (SSC) region of 18,013 bp. The cp genome contains 128 complete genes, including 83 protein-coding genes (83 PCGs), eight ribosomal RNA genes (four rRNAs), and 37 tRNA genes (37 tRNAs). Most genes occur in a single copy, while 16 genes occur in double, including four rRNAs, seven tRNAs (trnA-UGC, trnI-CAU, trnI-GAU, trnL-CAA, trnN-GUU, trnR-ACG, and trnV-GAC), and five PCGs (rps7, rpl2, rpl23, ndhB, and ycf2). The overall AT content of cp DNA is 63.6%, and the corresponding values of the LSC, SSC, and IR regions are 65.8%, 71.0%, and 57.0%.

In order to further clarify the phylogenetic position of *L. apetalum*, plastome of nine representative Brassicaceae species was obtained from NCBI to reconstruct the plastome phylogeny, with *Tarenaya hassleriana* as an outgroup. All the sequences were aligned using MAFFT v.7.313 (Katoh and Standley 2013) and maximum-likelihood phylogenetic analyses were conducted using RAxML v.8.2.11 (Stamatakis 2014). The phylogenetic tree shows that the species of Brassicaceae were divided into two subclades. All *Lepidium* species and *Capsella grandiflora* clustered together, while Remian species clustered in another clade, with *L. apetalum* clustered together with *L. sativum* and *L. virginicum* (Figure 1).

**Figure 1.**
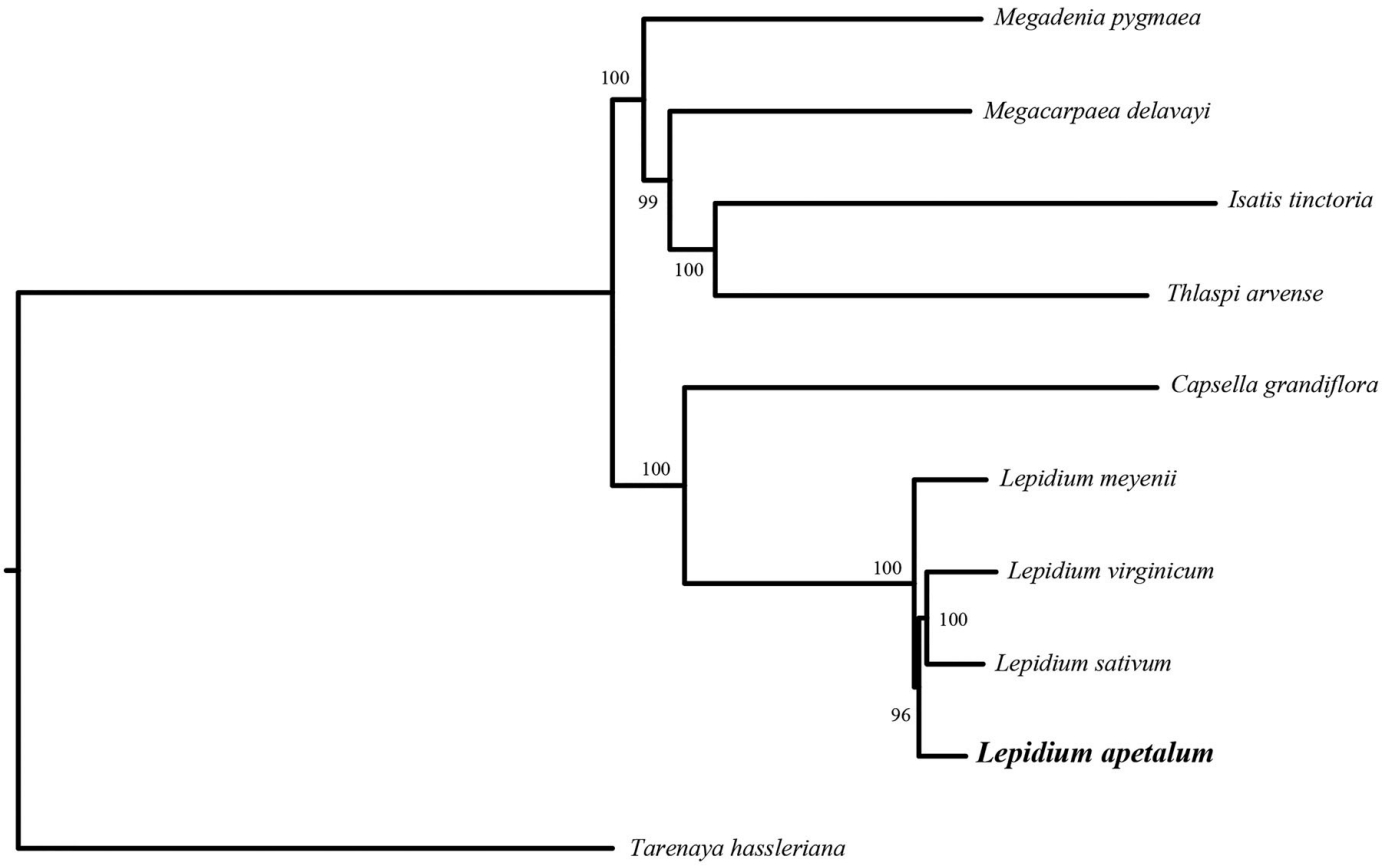
Phylogenetic relationships of Brassicaceae species using whole chloroplast genome. GenBank accession numbers: *Capsella grandiflora* (NC_028517), *Isatis tinctoria* (NC_028415), *Lepidium apetalum* (MT588298), *Lepidium meyenii* (MT430983), *Lepidium sativum* (NC_047178), *Lepidium virginicum* (AP009374), *Megadenia pygmaea* (NC_034357), *Megacarpaea delavayi* (KX886349), *Thlaspi arvense* (NC_034362), and *Tarenaya hassleriana* (KX886354).

## Data Availability

The data that support the findings of this study are openly available in GenBank of NCBI at https://www.ncbi.nlm.nih.gov, reference number MT588298.
